# Evaluation of the Clinical Value of the Serological Markers CD276 and DKK3 in Gastric Cancer: A Case–Control Study

**DOI:** 10.3390/diagnostics16060840

**Published:** 2026-03-12

**Authors:** Cosmina Fugărețu, Valeriu Șurlin, Catalin Misarca, Ana-Maria Ciurea, Stefan Patrascu, Dumitru Sandu Ramboiu, Mihail Virgil Boldeanu, Adina Turcu-Stiolica, Stiliani Laskou, Cicerone Catalin Grigorescu

**Affiliations:** 1 Doctoral School, University of Medicine and Pharmacy of Craiova, 200349 Craiova, Romania; comanescu_cosmina@yahoo.com (C.F.); vsurlin@gmail.com (V.Ș.); 2 1st General Surgery Department, Brașov County Emergency Clinical Hospital, 500326 Brașov, Romania; 3 Faculty of General Medicine Brașov, Transilvania University, 500036 Brașov, Romania; 4 1st Clinic of Surgery, Emergency Hospital of Craiova, 200642 Craiova, Romania; stef.patrascu@gmail.com (S.P.); sandu_r@yahoo.com (D.S.R.); 5 Faculty of General Medicine Craiova, University of Medicine and Pharmacy of Craiova, 200642 Craiova, Romania; 6 Academy of Romanian Scientists, 050045 Bucharest, Romania; 7 Department of Oncology, Emergency County Hospital, University of Medicine and Pharmacy of Craiova, 200349 Craiova, Romania; 8 Department of Immunology, University of Medicine and Pharmacy of Craiova, 200349 Craiova, Romania; boldeanumihailvirgil@yahoo.com; 9 Department of Pharmacoeconomics, University of Medicine and Pharmacy of Craiova, 200349 Craiova, Romania; adina.turcu@umfcv.ro; 10 Department of Health Economics and Outcomes Research, “Iuliu Hatieganu” University of Medicine and Pharmacy Cluj-Napoca, 400012 Cluj-Napoca, Romania; 11 3rd Surgical Department, University General Hospital of Thessaloniki “AHEPA”, School of Medicine, Faculty of Health Sciences, Aristotle University of Thessaloniki, 54621 Thessaloniki, Greece; stelaskou@gmail.com; 12 Department of Anatomy, Faculty of Medicine, “Vasile Goldiș” Western University of Arad, 310414 Arad, Romania; grigorescu.cicerone@uvvg.ro

**Keywords:** gastric cancer, CD276, B7-H3, DKK3, biomarker, diagnosis, ROC curve

## Abstract

**Background**: Gastric cancer (GC) remains a global health challenge, with high mortality rates often linked to late-stage diagnosis. Novel, non-invasive biomarkers are urgently needed to improve the detection and prognosis of this malignant pathology. This study aimed to evaluate the diagnostic and prognostic utility of serum Cluster of Differentiation 276 (CD276) and Dickkopf Related Protein 3 (DKK3) in patients with GC. **Methods**: In this case–control study, serum levels of CD276 and DKK3 were quantified in 40 GC patients and 40 age-matched healthy controls. The diagnostic performance of each marker and their combination was assessed using Receiver Operating Characteristic (ROC) curve analysis. Correlations between biomarker levels and clinicopathological features were evaluated using Spearman’s correlation. The Kaplan–Meier method and the Cox Proportional Hazards Regression Model were used to assess survival. **Results**: Serum CD276 levels were found to be significantly elevated in GC patients compared to healthy controls (median 60.06 vs. 18.71 units, *p* < 0.001). Conversely, serum DKK3 levels were significantly suppressed in the GC group (median 92.47 vs. 121.02 units, *p* < 0.001). In ROC analysis, CD276 demonstrated excellent diagnostic accuracy as a standalone biomarker (AUC: 0.836). DKK3 showed independent diagnostic value (AUC: 0.792), but adding DKK3 to CD276 did not provide statistically significant incremental benefit (DeLong’s *p* = 0.443). Survival analysis was underpowered due to limited events and short follow-up duration. **Conclusions**: In patients with predominantly locally advanced gastric cancer, CD276 can be a primary diagnostic marker, and the addition of DKK3 does not demonstrate a statistically significant improvement but may provide complementary information. Performance in early-stage disease requires validation in future studies. The opposing dysregulation of these markers, reflecting immune checkpoint activation (CD276) and tumor suppressor loss (DKK3), provides a robust and synergistic noninvasive signature. To assess the prognostic value of these two markers, studies involving a larger number of patients and a longer follow-up period are needed.

## 1. Introduction

Although the overall incidence of gastric cancer (GC) has decreased significantly over the last decade, it remains one of the malignant pathologies with a poor prognosis, with an overall patient survival rate of less than 10 months after diagnosis [1].

GC is a highly heterogeneous disease, so although some patients are in similar tumor stages and have similar tumors from an anatomopathological perspective, their prognosis and survival may differ [2]. All of this has led to the need to discover new molecular biomarkers that are useful in the diagnosis and prognosis assessment of GC patients, as clinical and anatomopathological staging are no longer sufficient [3].

Cluster of Differentiation 276 (CD276) and Dickkopf-3 (DKK3) may be such markers.

CD276 is also called B7-H3 and is part of the B7 protein family, with between 20 and 27% of its amino acids identical to the other 10 proteins in the family [4]. It is encoded by a gene located on chromosome 15q24.1 and acts in tumor pathology through immune and non-immune pathways [5,6,7]. Thus, CD276 inhibits peritumoral infiltration with inflammatory cells, suppresses T and natural killer (NK) cell function, and leads to decreased cytokine release, thereby evading antitumor immune defense [5]. The reduction in T and NK cell activity within the tumor microenvironment has also been reported in other malignancies, including lung cancer, underscoring the role of immune suppression in tumor progression [8].

Through non-immune pathways, CD276 promotes the migration, invasion, and metastasis of malignant cells [5]. This is possible by blocking cell cycle checkpoints via the phosphatidylinositol 3-kinase and protein kinase B pathway and by altering lipid and glucose metabolism in malignant cells [9]. Furthermore, systemic inflammatory–nutritional status has been shown to significantly influence cancer prognosis, highlighting the complex interplay between tumor biology and host immune response [8].

The soluble form of B7-H3 (sB7-H3) is generated by the cleavage of the transmembrane protein by metalloproteases and can interact with specific receptors, thus exerting effects on T cells, and increased serum levels of this molecule are associated with an unfavorable prognosis in malignant diseases [5,10].

The presence of B7-H3 or CD276 has been frequently studied in known cases of colorectal, lung, or breast cancer and much less frequently in cases diagnosed with GC [11].

On the other hand, DKK3 is a protein in the Dickkopf (DKK) family that has multiple roles in humans, but in malignant pathology it plays a tumor-suppressing role through its involvement in both cell proliferation and cell apoptosis [12].

The gene encoding the DKK3 protein is located on chromosome 11p15.3, and it has been found that promoter hypomethylation is common in tumor tissue, resulting in decreased expression of the DKK-3 glycoprotein during carcinogenesis [13,14].

It appears that the inhibitory role that DKK3 plays in malignant diseases can be achieved both by inhibiting the Wnt pathway and by its interaction with the epidermal growth factor (EGF) receptor, as demonstrated “in silico” [15].

Furthermore, DKK3 expression at the tumor level leads to increased intercellular adhesion by increasing E-cadherin expression and decreasing the mesenchymal marker Snail-1. Thus, DKK3 appears to play a role in epithelial–mesenchymal transition by decreasing cell motility and the invasive capacity of tumor cells [16,17,18,19].

In this study, we aimed to compare the serum levels of DKK3 and CD276 determined in patients diagnosed with GC at various stages of the disease with those determined in a group of healthy individuals in order to assess the diagnostic and prognostic role of these markers in gastric cancer.

One of the general objectives is to identify a correlation between the serum values of the aforementioned markers and the tumor stage of malignant gastric disease, the depth of tumor invasion (T), the presence of lymph node invasion (N), the number of invaded lymph nodes, the presence of perivascular and perineural invasion, carcinoembryonic antigen (CEA), and carbohydrate antigen 19-9 (CA 19-9) values determined at the time of diagnosis, as well as weight loss, together with the status of tumor resection margins (positive or negative) and peritoneal cytology.

The assessment of Overall Survival (OS) and Disease-Free Survival (DFS) is another objective of the study, along with the presence of metastases at diagnosis and the occurrence of death during follow-up.

## 2. Materials and Methods

### 2.1. Patient Selection

To achieve the above-mentioned objectives, we formed two groups of patients. In the first group, called Group 1, we included consecutively enrolled patients diagnosed with gastric cancer who were treated in two surgical departments, namely the 1st General Surgery Department of the Brașov County Emergency Clinical Hospital (SCJUBV) and the 1st Clinic of Surgery of the Emergency Hospital of Craiova (SCJUCV) between September 2023 and February 2025. In the second group, called Group 2 or the control group, we included people who had gastric cancer ruled out by undergoing an esophagogastroduodenoscopy (EGD) on an outpatient basis and who had no personal history of other malignant diseases.

The inclusion and exclusion criteria for participants are presented in Table 1.

The study was conducted in accordance with the Declaration of Helsinki and was approved by the Ethics Committee of the University of Medicine and Pharmacy in Craiova (no. 193/20 September 2023).

After forming the two groups, we extracted and analyzed a series of demographic, clinical, and paraclinical data using observation sheets, as well as test reports and histopathological results of resection specimens in the case of patients in Group 1.

Among the data we considered of interest were gender, age, background, weight loss, behaviors such as smoking or alcohol consumption, the existence of other associated pathologies, family history of cancer, date of diagnosis, and the existence of neoadjuvant chemotherapy treatment with specification of the therapeutic regimen.

Weight loss was calculated as the difference between body weight in the last 3–6 months and body weight determined preoperatively. Patients were considered chronic alcohol consumers if they reported drinking alcohol almost daily for months to years, often >40–60 g of pure alcohol/day for men and >20–40 g of pure alcohol/day for women [20].

Also, to maintain anonymity, the data were anonymized.

The presence of tumor cells in the peritoneal fluid was identified by cytological analysis of approximately 5 mL of peritoneal fluid collected at the beginning of the surgical intervention after local lavage with approximately 100 mL of physiological serum.

For patients diagnosed with gastric cancer, we also extracted other information obtained after macroscopic and microscopic examination of the resection specimens, such as tumor size, the depth of tumor extension, the number of lymph nodes identified with specification of the number of tumor-invaded lymph nodes, and the presence of perineural and perivascular invasion.

TNM staging was defined according to the 8th edition proposed by the American Joint Committee on Cancer (AJCC) [21]. Histological subtypes of gastric cancer were defined based on the 2019 World Health Organization (WHO) classification of digestive system tumors [22].

To assess short-term survival, patients were contacted by telephone at 3, 6, and 12 months.

### 2.2. Sample Collection

Each patient provided one tube without additives, containing approximately 5 mL of venous blood (Becton, Dickinson and Company, Franklin Lakes, NJ, USA). The collection was performed before surgery for GC participants in Group 1 and after EGD for those in Group 2. After collection, samples were processed according to standard protocols: centrifuged at 3000× *g* for 10 min in a Hermle centrifuge (Hermle AG, Gosheim, Baden-Württemberg, Germany) within 4 h of collection and after clotting. The serum from the tube was aliquoted into pre-labeled, sealed vials to prevent contamination and stored at temperatures between −20 °C and −80 °C. Freeze–thaw cycles were avoided. Frozen serum was thawed at room temperature before analysis. Immunological tests used these aliquots.

### 2.3. Serum Determination of CD276 and DKK3

We determined the serum levels of CD276 and DKK3 using the sandwich enzyme immunoassay method and the Enzyme-Linked Immunosorbent Assay (ELISA) Kits from Elabscience (Elabscience, Houston, TX, USA):

- Human CD276 (Cluster of Differentiation 276): Cat. No.: E-EL-H6230; product Link: https://file.elabscience.com/Manual/elisa_kits/E-EL-H6230-Elabscience.pdf (accessed on 10 November 2025); Sensitivity 0.08 ng/mL; Detection Range 0.16–10 ng/mL; Specificity: This kit recognizes Human CD276 in samples. No significant cross-reactivity or interference between Human CD276 and analogues was observed; Repeatability: Coefficient of variation is <10%.
- Human DKK3 (Dickkopf Related Protein 3): Cat. No.: E-EL-H6110; product Link: https://file.elabscience.com/Manual/elisa_kits/E-EL-H6110-Elabscience.pdf (accessed on 10 November 2025); Sensitivity 0.47 ng/mL; Detection Range 0.78–50 ng/mL; Specificity: This kit recognizes Human DKK3 in samples. No significant cross-reactivity or interference between Human DKK3 and analogues was observed; Repeatability: Coefficient of variation is <10%.

These kits contain a microtiter plate pre-coated with a specific antibody for CD276, and the second kit contains a microtiter plate pre-coated with a specific antibody for DKK3. We followed the manufacturer’s instructions and, after pipetting the samples onto the microtiter plate, added the biotin-conjugated antibodies specific for human CD276 and DKK3, respectively. Subsequently, we added horseradish peroxidase (HRP)-conjugated avidin to each well of the microplate, which was incubated. We then added TMB, which caused a color change only in the wells containing human CD276 and DKK3, respectively. We then added the sulfuric acid solution, and the colorimetric change was quantified spectrophotometrically at 450 nm ± 10 nm. The concentrations of human CD276 and human DKK3 in the samples were evaluated by comparing their optical densities (OD) with the standard curve. To achieve this, we used an optical analyzer (Asys Expert Plus UVG020 150 Microplate Reader, ASYS Hitech GmbH, Eugendorf, Austria) at 450 nm.

### 2.4. Statistical Analysis

All statistical analyses were performed using R Statistical Software for Windows version (v4.5.0, R Foundation for Statistical Computing, 2025, Vienna, Austria). A two-sided *p*-value of < 0.05 was considered statistically significant.

- Descriptive Analysis

Continuous variables (e.g., DKK3, CD276, age, and tumor size) were tested for normal distribution using the Kolmogorov–Smirnov test. Data were summarized as the mean ± standard deviation (SD) for normally distributed variables or the median [interquartile range (IQR)] for non-normally distributed data. Categorical variables (e.g., sex, histopathological type, resection margins, and smoking status) were reported as absolute frequencies (*n*) and percentages (%).

b. Comparison of biomarker levels

To compare serum levels of CD276 and DKK3 between the GC patient group, Group 1, and the healthy control group, Group 2, the Independent Samples t-test was utilized for normally distributed data, and the Mann–Whitney U test was used for non-normally distributed data.

The diagnostic performance of CD276 and DKK3 was evaluated with Receiver Operating Characteristic (ROC) curve analysis using binary logistic regression: the outcome was Gastric cancer diagnosis (1) vs. control (0), and the predictors were CD276 and DKK3 serum levels (both continuous). The Area Under the Curve (AUC), along with its 95% Confidence Interval (CI), was calculated to assess the discriminative power. The optimal cut-off value for each biomarker was determined using the Youden index (maximum of sensitivity + specificity − 1), which was then used to categorize patients into high- and low-level groups. Sensitivity, specificity, positive predictive value (PPV), and negative predictive value (NPV) were subsequently reported for the optimal cut-offs.

To determine whether the combined CD276 + DKK3 model provided statistically significant improvement over individual biomarkers, we performed pairwise comparison of correlated ROC curves using DeLong’s test. This non-parametric method is specifically designed for comparing AUC values derived from the same dataset.

To address potential optimistic bias resulting from deriving and evaluating cutoff values in the same dataset, we performed bootstrap internal validation with 1000 replications. For each bootstrap iteration, we randomly resampled the original dataset with replacement (*n* = 80, maintaining the 40:40 case–control ratio), determined optimal cutoff values using Youden’s index on the bootstrap sample, and calculated diagnostic performance metrics (AUC, sensitivity, specificity). These bootstrap-derived cutoffs were then applied to the original dataset to calculate test performance. Optimism was quantified as the mean difference between bootstrap-sample performance and original-data performance across all iterations. Optimism-corrected estimates were calculated by subtracting the mean optimism from apparent (original) performance estimates. Bootstrap 95% confidence intervals were derived from the 2.5th and 97.5th percentiles of the bootstrap distribution. All bootstrap analyses were performed using the pROC package in R version 4.5.2 (R Foundation for Statistical Computing, Vienna, Austria), with random seed set to 123 for reproducibility.

c. Correlation and association analysis

The association between serum CD276 and DKK3 levels and other continuous clinicopathological variables (e.g., CEA, CA 19-9 levels, tumor size, lymph node ratio) was determined using Pearson’s correlation coefficient (r) for normally distributed data and Spearman’s rank correlation coefficient (rho) for non-normally distributed data.

To assess the relationship between biomarker levels and categorical variables (e.g., TNM stage, G-grade, perineural/perivascular invasion, *H. pylori* status), the Mann–Whitney U test (for two groups) or the Kruskal–Wallis H test (for more than two groups) was employed. If the Kruskal–Wallis test was significant, post hoc analysis with Dunn’s test was performed to identify specific group differences.

d. Survival analysis

The primary endpoints for prognostic analysis were OS and DFS. Survival time was calculated from the date of surgery until the date of death (for OS) or the date of recurrence (for DFS) or the date of last follow-up (for DFS). For univariate survival analysis, survival curves for patient subgroups, dichotomized based on the optimal DKK3 and CD276 cut-offs, were generated using the Kaplan–Meier method. Differences in survival curves were compared using the log-rank test. For multivariable survival analysis, a Cox Proportional Hazards Regression Model was used to assess the independent prognostic value of CD276 and DKK3 after adjusting for established clinicopathological factors (e.g., pT stage, pN stage, resection margins, histopathological type). The results were reported as Hazard Ratios (HRs) and their 95% CIs. The proportional hazards assumption was visually checked using Schoenfeld residuals.

## 3. Results

### 3.1. Patient Characteristics

**Figure 1:**
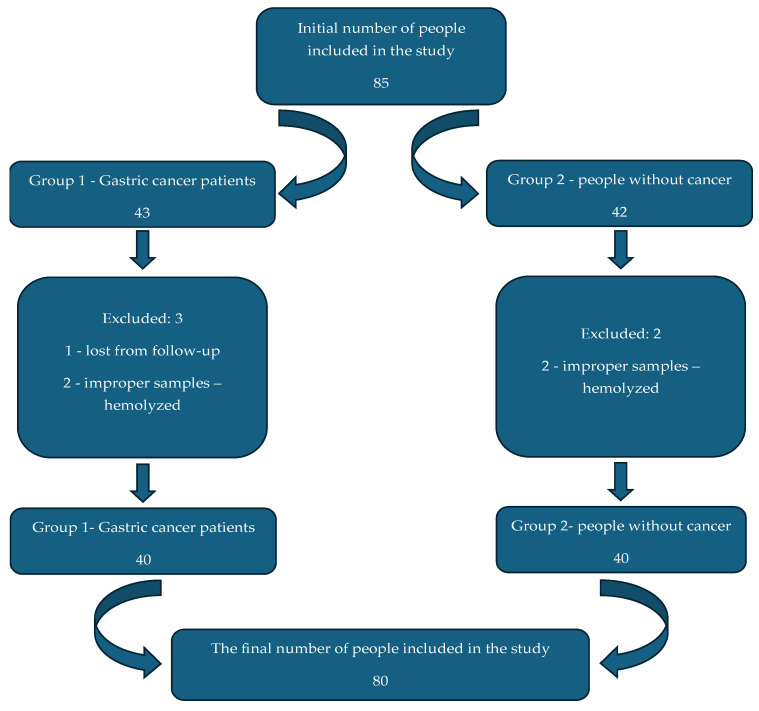
Final number of individuals included in the study.

Initially, we included 85 people in the study, of whom 43 were placed in Group 1, being patients diagnosed with gastric cancer, and 42 people were placed in Group 2, in whom gastric cancer was excluded by performing an EGD. We ultimately excluded 3 individuals from Group 1 because one was lost to follow-up, and the samples from the other two individuals were unsuitable due to hemolysis. Two individuals were excluded from Group 2 because their serum samples were unsuitable. Thus, in the end, 40 patients were included in each of the two groups (Figure 1).

The baseline patient characteristics in Group 1 and detailed clinicopathological features are summarized in Table 2. The mean age of the group was 65.83 ± 8.47 years, with a median age of 66 years (IQR: 61.25–70.75). The majority of the patients were male (77.5%) and resided in urban areas (55%). A high percentage reported a history of smoking (65%), but equal chronic alcohol consumption (50%). The group also presented with significant disease burden, indicated by a mean preoperative weight loss of 0 6.18 ± 3.91 kg. The predominant tumor location was antral (67.5%). The histopathological assessment revealed a strong majority of intestinal-type adenocarcinoma (85%), with a smaller fraction of signet ring cell carcinoma (15%).

The disease was largely locally advanced at diagnosis: 65% of patients had T3 or T4a disease (32.5% T3, 32.5% T4a). A high proportion exhibited extensive nodal disease, with 50% classified as N3a or N3b. The majority of patients presented with Stage III or IVA disease (62.5%). High rates of vascular and neural invasion were noted: perivascular invasion in 67.5% and perineural invasion in 57.5%. Positive peritoneal fluid cytology was observed in 42.5% of patients.

The majority of gastric cancer patients presented with locally advanced disease, with 72.5% diagnosed as TNM Stage III or IVA. This distribution reflects typical presentation in our region where screening is not established. The limited number of early-stage cases (Stage I: *n* = 4; Stage II: *n* = 11) precluded meaningful stage-stratified biomarker analysis. Therefore, diagnostic accuracy metrics reported herein primarily reflect discriminatory capacity between locally advanced gastric cancer and healthy controls, rather than early-stage detection performance.

The main characteristics of the individuals included in Group 2 are presented in Table 3. Controls were categorized as having gastritis (*n* = 15, 37.5%) or normal gastric mucosa (*n* = 25, 62.5%) for stratified analyses to assess the influence of inflammatory status on CD276 levels.

### 3.2. Differential Serum Expression of CD276 and DKK3

**Figure 2:**
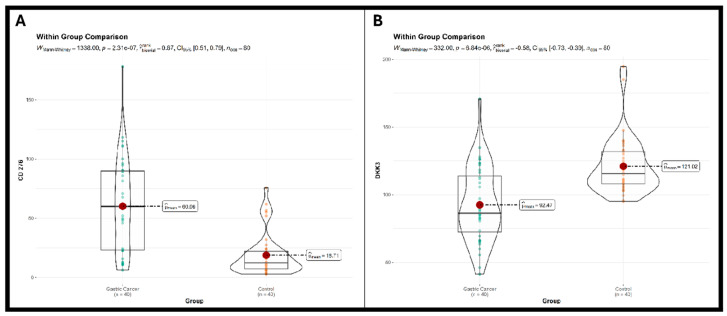
Differential serum expression between the gastric cancer group (Group 1) and control group (Group 2). (**A**) For marker CD276. (**B**) For marker DKK3. Mann–Whitney U test. Data are presented as raincloud plots combining three visualizations: (1) box plots showing mean (red dot) and interquartile range (IQR; box edges = 25th and 75th percentiles), (2) violin plots showing distribution shape (width = density of data points), and (3) individual data points (dots) representing each patient’s measurement. Dashed lines connect mean to its numerical label.

Serum concentrations of the two candidate biomarkers, CD276 and DKK3, were compared between the GC cohort, Group 1, and the control group, Group 2. Both markers exhibited highly significant differential expressions between the groups, underscoring their potential as diagnostic biomarkers. The median serum concentration of CD276 was significantly higher in patients with GC (median = 60.06 units) compared to the healthy control group (median = 18.71 units). This difference was highly statistically significant, as determined by the Mann–Whitney U test (*p* < 0.0001). The magnitude of the effect was large, with a rank biserial correlation coefficient of r = 0.67, as in Figure 2A. In contrast to CD276, the median serum concentration of DKK3 was significantly lower in the GC cohort (median = 92.47 units) compared to the healthy controls (median = 121.02 units). This inverse relationship was also highly statistically significant (*p* < 0.0001). The effect size for DKK3 was substantial, with a rank biserial correlation coefficient of r = −0.58, as in Figure 2B.

### 3.3. Diagnostic Performance of Individual and Combined Biomarkers

**Figure 3:**
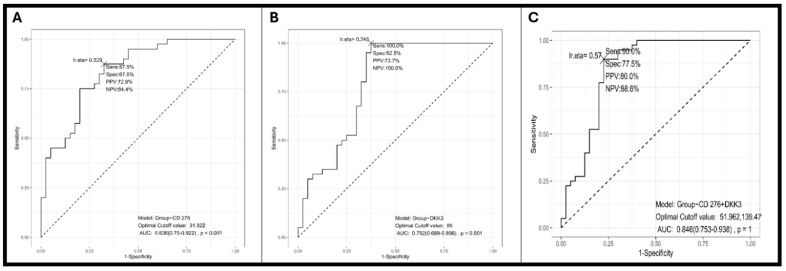
ROC curve analysis for CD276 (**A**), DKK3 (**B**), and CD276 and DKK3 (**C**). The area under the ROC curve (AUC) summarizes overall diagnostic accuracy: AUC = 0.5 indicates no discriminatory ability (equivalent to random chance, represented by the diagonal dashed reference line); AUC = 1.0 indicates perfect discrimination. Generally, AUC values of 0.70–0.80 are considered acceptable, 0.80–0.90 good, and >0.90 excellent diagnostic performance.

The diagnostic accuracy of each marker and their combination was assessed using ROC curve analysis, as shown in Figure 3. The area under the curve (AUC) for CD276 was 0.836 (95% CI: 0.75), indicating good discriminatory power. At the optimal cutoff value of 31.9 units, CD276 demonstrated a sensitivity of 87.5% and a specificity of 67.5%, as shown in Figure 3A (worse prognosis at CD276 levels > 31.9 units). The AUC for DKK3 was 0.792 (95% CI: 0.68), also indicating good diagnostic capability, as shown in Figure 3B. At its optimal cutoff of 95 units, DKK3 achieved a sensitivity of 100.0% but a lower specificity of 62.5% (better survival for serum DKK3 level ≥ 95 units). Combining the two markers into a single diagnostic model yielded the highest accuracy. The combination model achieved an AUC of 0.846 (95% CI: 0.753–0.938), as shown in Figure 3C. At the determined optimal cutoff values, the combined panel demonstrated a sensitivity of 90.0% and a specificity of 77.5%, resulting in a Positive Predictive Value (PPV) of 80.0% and a Negative Predictive Value (NPV) of 88.6%, as shown in Figure 3C.

Adding DKK3 to CD276 did not provide statistically significant incremental benefit (DeLong’s z = 767, *p* = 0.443). These findings indicate that CD276 is the primary diagnostic biomarker, with the combined panel showing a numerical trend toward improvement but lacking statistical significance for the incremental benefit of adding DKK3 in this cohort of 40 patients per group.

To address potential optimistic bias from deriving and evaluating cutoffs in the same dataset, we performed bootstrap internal validation with 1000 replications. For CD276, bootstrap validation revealed minimal optimism. The corrected AUC of 0.837 was virtually identical to the apparent AUC of 0.836, with optimism of only −0.001, indicating the model is not overfit. Sensitivity decreased from 80.0% to 77.4% (optimism: 2.6%), and specificity from 75.0% to 72.1% (optimism: 2.9%). Despite these corrections, CD276 maintained good-to-excellent diagnostic performance (corrected AUC > 0.80).

For DKK3, bootstrap validation encountered statistical instability due to the optimal cutoff producing 100% specificity in the apparent performance, which proved non-sustainable in bootstrap resampling. This suggests the DKK3 optimal cutoff is sensitive to the specific composition of the dataset.

The minimal optimism observed for CD276 (AUC optimism of −0.001, sensitivity and specificity optimism of 2.6% and 2.9%) is well within expected ranges for studies of this sample size and indicates that the diagnostic performance is robust and not artificially inflated by overfitting to the derivation dataset.

To assess the influence of inflammatory conditions on CD276 diagnostic specificity, we performed stratified analyses comparing CD276 levels across gastric cancer patients, controls with gastritis, and controls without gastritis. ROC analysis was performed separately for cancer versus controls stratified by gastritis status (Table 4). Cancer versus controls without gastritis (*n* = 40 vs. 25) yielded AUC of 0.879 (95%CI: 0.8–0.96) with sensitivity of 84% and specificity of 80%, representing diagnostic performance in the absence of inflammatory confounding. Cancer versus controls with gastritis (*n* = 40 vs. 15) yielded AUC of 0.765 (95% CI: 0.64–0.89) with sensitivity of 67.5% and specificity of 86.7%, representing the clinically relevant scenario of discriminating cancer from benign inflammatory conditions. The original analysis comparing cancer to all controls (AUC 0.837) represents an intermediate performance reflecting the mixed control group composition (37.5% with gastritis, 62.5% without).

### 3.4. Correlation of Serum DKK3 and CD276 Levels with Clinicopathological Factors

**Figure 4:**
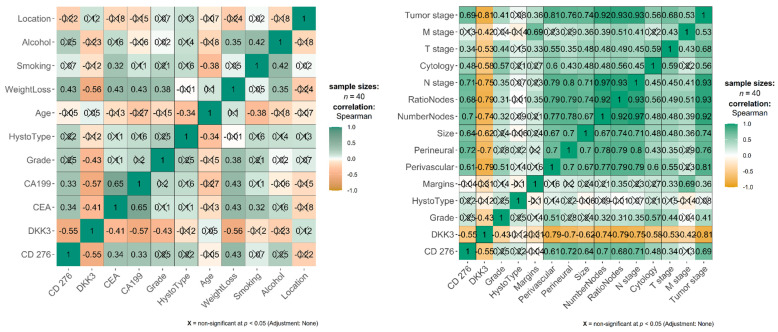
Spearman correlation analysis evaluating the relationship between serum biomarker levels (CD276 and DKK3) and various clinicopathological features in Group 1. Color scale represents the positive correlation (green) and negative correlation (orange), color intensity shows the correlation strength (darker is stronger: perfect negative, rho = −1.0, dark orange and perfect positive, rho = +1.0, dark green). Numbers are Spearman’s rho, ρ, coefficients.

A Spearman correlation analysis was performed to evaluate the relationship between serum biomarker levels (CD276 and DKK3) and various clinicopathological features and classical tumor markers in the GC cohort, as shown in Figure 4.

The analysis of serological and systemic variables revealed a strong, opposing relationship between the two novel markers and established clinical metrics. A highly significant, strong negative correlation was observed between CD276 and DKK3 (ρ = −0.55, *p* < 0.001), confirming their antagonistic roles in the disease process.

The markers also showed distinct associations with systemic disease burden and established serological metrics: DKK3 was significantly and inversely correlated with weight loss (ρ = −0.56, *p* < 0.001), indicating that lower DKK3 levels are associated with increased systemic catabolism. Also, CD276 showed a positive and statistically significant correlation with weight loss (ρ = 0.43, *p* = 0.006). DKK3 demonstrated a significant negative correlation with both CEA (ρ = −0.41, *p* = 0.009) and CA19-9 (ρ = −0.57, *p* = 0.0001). At the same time, CD276 showed a statistically significant correlation with both CEA (ρ = 0.34, *p* = 0.03) and CA19-9 (ρ = 0.33, *p* = 0.04).

CD276 did not exhibit a significant positive correlation with grade (ρ = 0.25, *p* = 0.13). Correspondingly, DKK3 showed a significant negative correlation (ρ = −0.43, *p* = 0.006) with grade, meaning higher DKK3 levels are associated with poorer tumor differentiation.

DKK3 showed a significant inverse correlation with the number of invaded nodes and the ratio of metastatic lymph nodes (ρ = −0.74, *p* < 0.001, and ρ = −0.79, *p* < 0.001, respectively), linking its depletion to a higher metastatic lymph node burden. Correspondingly, CD276 showed a significant direct correlation with the number of invaded nodes and the ratio of metastatic lymph nodes (ρ = 0.71, *p* < 0.001) and (ρ = −0.68, *p* < 0.001), respectively, linking its depletion to a higher metastatic lymph node burden.

**Figure 5:**
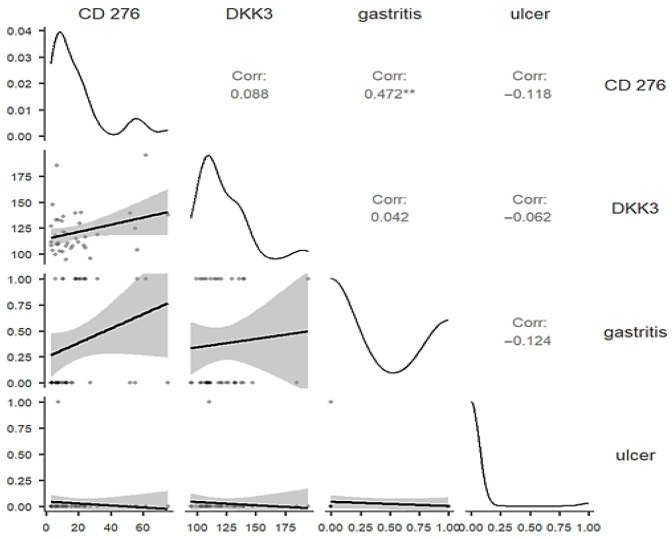
Correlation analysis evaluating the relationship between serum biomarker levels (CD276 and DKK3) and the presence of gastritis and ulcer in the control group, Group 2. ** *p* < 0.01.

To specifically assess whether the dysregulation of CD276 and DKK3 is linked to benign pathology, we analyzed the correlation between biomarker levels and the presence of co-occurring gastric ulcers or gastritis within the control group, Group 2. CD276 demonstrated a statistically significant positive correlation with the presence of gastritis, as in Figure 5 (rho = 0.472, *p* = 0.002), indicating that the systemic CD276 concentration increases with the presence of benign gastric inflammation. Serum CD276 levels did not show a statistically significant correlation with the presence of ulcer (rho = −0.118, *p* = 0.469). DKK3 showed no statistically significant correlation with either gastritis (rho = 0.042, *p* = 0.795) or gastric ulcer (rho = −0.062, *p* = 0.702).

### 3.5. Survival Analysis

The univariate prognostic value of the serum CD276 level and DKK3 were assessed using the Kaplan–Meier method with the log-rank test for OS. The non-significant *p*-value of 0.999 for CD276 and the dramatically unstable Hazard Ratio (HR ≈ 3.4 × 10^8^) indicate that the Cox Proportional Hazards model failed due to perfect separation. This occurred because the low CD276 group (Group 0) had zero events (deaths). When one group has 100% survival during the observed period, the proportional hazards assumption is violated, rendering the HR and *p*-value unreliable. Despite the magnitude of the HR for DKK3, the *p*-value (0.291) is not statistically significant. Additionally, the 95% CI for the HR (0.37–27.25) is extremely wide and crosses 1.0, confirming that DKK3, in isolation, is not an independent prognostic factor for OS in this cohort.

The multivariable model remained unstable, yielding a very high HR for CD276, confirming the model failure observed in the univariate analysis due to perfect separation (zero events in the reference group). The Cox regression model is built on only 6 events (a stable Cox regression model requires a minimum of 10–15 events (deaths) per variable included). M Stage (M1 vs. M0) is the single strongest prognostic factor in all of oncology. The fact that it is not statistically significant (*p* = 0.762) is definitive proof that the model is unstable and has failed.

A Cox proportional hazards model was performed to assess the impacts of CD276, DKK3, and key clinicopathological factors on DFS. In the univariate analysis, several variables showed extremely high, unstable hazard ratios (HRs) and non-significant *p*-values, indicative of model instability (likely due to zero events in a reference group), as in Table 4. Conversely, T stage (T3, T4a, T4b) and M stage emerged as significant predictors in the univariate model: for T stage (T3 vs. T1): HR = 41 × 10^6^, *p* < 0.001 and M stage (M1 vs. M0): HR = 81.53 (95% CI, 1.1–6061.86, *p* = 045).

A multivariable model was constructed to test the independent prognostic value of the biomarkers alongside established clinical factors (T stage, ratio of nodes, and M stage). Neither was the biomarker a significant independent predictor of DFS, as shown in Table 5.

## 4. Discussion

This case–control study evaluated the diagnostic utility of serum CD276 and DKK3, individually and in combination, in patients with predominantly locally advanced gastric cancer. Our key findings demonstrate that serum CD276 is a robust standalone diagnostic biomarker with good-to-excellent performance (bootstrap-corrected AUC 0.837, sensitivity 77.4%, specificity 72.1%). DKK3 showed independent diagnostic value (apparent AUC 0.792), though with greater sensitivity to overfitting. The combined panel achieved numerically higher performance (AUC 0.846) but did not demonstrate statistically significant improvement over CD276 alone (DeLong’s *p* = 0.443), likely due to limited statistical power in our cohort of 40 patients per group.

The characteristics of the GC patients reflect a cohort with a considerable burden of locally advanced disease, which is unfortunately common in regions where screening programs are not widely established. The high mean age (65.83 years), male predominance (77.5%), and high prevalence of smoking and alcohol consumption align with known epidemiological risk factors for gastric cancer [23,24].

CD276 demonstrated consistent diagnostic performance across both apparent and bootstrap-corrected analyses. The significant elevation of serum CD276 in gastric cancer patients (median 60.06 vs. 18.71 units, *p* < 0.001) reflects the well-established upregulation of membrane-bound CD276 in gastric cancer tissue and its subsequent shedding via metalloprotease cleavage [5,10].

An important consideration is the stage distribution of our cohort. With 72.5% Stage III/IVA disease, our results demonstrate the CD276/DKK3 panel can effectively discriminate between locally advanced gastric cancer and healthy controls, aligning with our primary diagnostic objective. Our findings should be interpreted as proof-of-concept that CD276/DKK3 dysregulation can be detected in serum and that the combined panel offers superior discriminatory capacity. The present study establishes biological plausibility and technical feasibility but does not constitute validation for clinical diagnostic use, particularly for early-stage detection. These directional changes—CD276 elevation and DKK3 downregulation—are consistent with the established functional roles of these proteins in cancer biology and highlight their potential utility as non-invasive serological biomarkers.

The role of these markers has also been studied in other malignant diseases. Thus, a recently published Chinese study concluded that serum B7-H3 (sB7-H3) values are significantly higher in patients with colorectal cancer compared to patients with benign colorectal diseases and healthy individuals [25]. These values determined in patients with colorectal cancer correlated weakly positively with serum CA50, CEA, and CA724 values, but also with B7-H3 expression in tumor tissue [25]. Our bootstrap-corrected AUC of 0.837 compares favorably to conventional tumor markers CEA and CA19-9 in gastric cancer, which typically achieve AUC values of 0.65–0.75.

Importantly, bootstrap internal validation revealed minimal optimism for CD276 (AUC optimism: −0.001; sensitivity optimism: 2.6%; specificity optimism: 2.9%). This remarkable stability demonstrates that CD276 diagnostic performance is robust and not artificially inflated by overfitting to the derivation dataset. The corrected AUC of 0.837 being virtually identical to the apparent AUC of 0.836 provides confidence that the reported metrics represent realistic estimates of true diagnostic accuracy within populations similar to our cohort.

DKK3 demonstrated independent diagnostic value with significant downregulation in gastric cancer patients (median 92.47 vs. 121.02 units, *p* < 0.001), confirming previous reports of DKK3 suppression in gastric cancer at both the tissue and serum levels. Regarding DKK3, a study published in 2021, which determined the presence of DKK3 in the serum of patients diagnosed with ovarian cancer as well as CD133 cells, revealed that DKK3 correlates negatively with the presence of circulating CD133+ and also that DKK3 has an inhibitory role on tumor cells by reversing the epithelial–mesenchymal transition. The conclusion of the study is that both serum DKK3 values and the number of circulating CD133+ cells may be prognostic markers in ovarian cancer [26].

Furthermore, certain studies have shown a significantly higher frequency of DKK3 gene methylation in free circulating DNA in blood or even urine in patients diagnosed with breast cancer or bladder cancer, respectively, compared to healthy individuals, thus concluding that it may represent a detectable tumor biomarker in blood and urine, respectively [13,27,28].

These markers play multiple roles in gastric cancer. Thus, in some studies, high levels of B7-H3 expression in the stromal compartment of gastric tumor tissue have been correlated with tumor location, Lauren staging, and tumor pT—the depth of tumor invasion [29]. It also appears that patients diagnosed with gastric cancer with high B7-H3 expression in tumor cells and tumor-associated fibroblasts have a lower survival rate [30]. On the other hand, the survival of gastric cancer patients who had increased expressions of B7-H3 and α-SMA (alpha-smooth muscle actin) in tumor-associated fibroblasts (CAFs) had a worse prognosis compared to other patients [30]. The absence of B7-H3 in tumor-associated fibroblasts led to reduced secretion of interleukin (IL)-6 and vascular endothelial growth factor (VEGF), as well as a decrease in the ability to migrate and invade cancer-associated fibroblasts (CAFs) [30].

During gastric carcinogenesis, from the onset of superficial chronic gastritis lesions to the onset of gastric cancer, a gradual increase in the expression of both CD276 and CD39 was observed [31]. These reached significantly higher levels in tumor tissue compared to precancerous lesions [31]. Furthermore, their high expression in tumor and peritumoral tissues was positively correlated with the depth of tumor invasion, lymph node invasion, and the presence of distant metastases, as well as with a poor prognosis [31].

Other studies have found that Helicobacter pylori strains associated with gastritis can induce increased B7-H3 expression in gastric epithelial cells and can modify T cell responses, thus promoting bacterial persistence [32]. In our study, the discovery of a significant positive correlation between CD276 and gastritis in healthy individuals (rho = 0.472, *p* = 0.002) is essential. CD276 (B7-H3) is an immune checkpoint, and its expression is known to be induced by pro-inflammatory cytokines such as IFN-ɣ and TNF-α.

On the other hand, it has been shown that, in gastric cancer, serum values of B7-H3 (sB7-H3) correlate with B7-H3 expression at the level of malignant cell membranes but not with its expression at the level of stromal cells, thus suggesting that the soluble form of B7-H3 originates from gastric tumor cells and not from the tumor stromal compartment [33].

The soluble form of B7-H3 can stimulate angiogenesis by increasing the expression of VEGF and IL-8, thus playing an active role in tumor progression [33,34].

In the literature, both serum CD276 (sB7-H3) values determined by ELISA and the expression of this marker at the cell membrane level were significantly higher in patients with gastric cancer compared to patients known to have gastritis or the group of healthy individuals [33]. Serum CD276 values were also significantly higher in patients with advanced gastric cancer compared to those in stage I/II and correlated with the depth of tumor invasion and the presence of lymph node invasion [33].

Our study also confirms the positive correlation between serum CD276 values determined in patients with gastric cancer and T-stage, the number of invaded nodes and the ratio of metastatic lymph nodes, and the presence of distant metastases. In addition to these parameters, the present study demonstrates a positive correlation between serum CD276 values and the presence of perineural invasion, the presence of malignant cells in peritoneal fluid, and preoperative weight loss.

Regarding serum DKK3 levels, studies have shown that gastric cancer patients with low DKK3 levels have more advanced TMN stages, more frequent lymph node invasion, and a lower survival rate compared to those with normal DKK3 levels [35].

Methylation of the promoter of the gene encoding DKK3 in patients with gastric cancer has been associated with a poorer prognosis and a more advanced stage of malignant disease compared to patients with the same condition but without this process [36].

A Chinese study published in 2019 found that DKK3 expression is much higher in tumor tissue samples from patients with gastric cancer without metastases compared to the expression of this glycoprotein in patients with metastatic disease [37]. The results show that low DKK3 expression by tumor cells correlates with their increased ability to migrate [37].

Some authors have revealed that low DKK3 expression in gastric tumor tissues correlates positively with more advanced tumor stage, the number of invaded lymph nodes, and the presence of lymphovascular and perineural invasion. Thus, the authors conclude that DKK3 can be considered a biomarker of lymph node invasion, and its preoperative determination together with CT examination can improve the assessment of lymph node invasion [38]. Our study revealed that low serum DKK3 levels correlate with the aforementioned clinical and pathological factors but also with other clinical and biological parameters such as the presence of malignant cells in the peritoneal fluid. It is known that low DKK3 expression is associated with low E-cadherin expression, which in turn plays an important role in intercellular adhesion [16,19].

On the other hand, in some studies, it has been shown that patients with low DKK3 expression had lower DFS and OS compared to those with increased DKK3 expression in tumor tissue, a fact that could not be confirmed by the serum DKK3 values determined by our study due to its limitations [38].

The individual performance of both CD276 (AUC: 0.836) and DKK3 (AUC: 0.792) indicates that each marker independently offers a respectable level of diagnostic utility. CD276 demonstrated a superior specificity (67.5%) compared to DKK3 (62.5%). As an immune checkpoint molecule shed by the tumor, elevated soluble CD276 likely serves as a direct, quantifiable reflection of tumor burden and active immune evasion. DKK3 provided exceptional sensitivity (100.0%). The loss of DKK3, a known tumor suppressor often silenced in malignancy, may represent an earlier and more pervasive systemic effect of carcinogenesis. Its high sensitivity suggests it may be highly effective at ruling out the disease (indicated by its high NPV). Serum CD276 demonstrates excellent diagnostic accuracy as a standalone biomarker. DKK3 shows independent diagnostic value but adding DKK3 to CD276 did not provide statistically significant incremental benefit (DeLong’s *p* = 0.443). Although the marginal increase in the overall AUC (0.846 vs. 0.836 for CD276 alone) was modest, the combination model significantly improved the specificity to 77.5% while maintaining high sensitivity at 90.0%. This synergy underscores the benefit of integrating markers that reflect different biological pathways: CD276 reflects the immunological environment and DKK3 reflects the tumor suppressor loss and Wnt pathway dysregulation. By combining these two distinct, yet complementary, biological signals, it fails to demonstrate a statistically significant improvement of the diagnostic.

A critical methodological consideration in biomarker studies is the potential for optimistic bias when cutoff values and performance metrics are derived and evaluated in the same dataset. Following rigorous bootstrap internal validation with 1000 replications, we found that CD276 exhibited minimal optimism across all performance metrics. The AUC optimism of −0.001 is essentially zero, and the sensitivity and specificity optimism of 2.6% and 2.9% respectively are well within expected ranges for studies of this sample size.

This minimal optimism is noteworthy for several reasons. First, it demonstrates that CD276 captures a strong, consistent biological signal that generalizes well across different resampled patient populations from our cohort. Second, it provides empirical evidence that our findings are not the result of overfitting or chance findings specific to our particular dataset composition. Third, it suggests that the optimal cutoff for CD276 is relatively stable and not highly sensitive to minor variations in patient composition.

A critical question for clinical translation is whether CD276 elevation is specific to malignancy or reflects inflammatory status more generally. Our stratified analyses directly address this concern and reveal important nuances regarding CD276’s diagnostic specificity. The biological basis for CD276 elevation in gastritis likely involves immune cell infiltration and inflammatory signaling in the gastric mucosa. B7-H3 is expressed on activated T cells, macrophages, and epithelial cells during inflammation, and functions as a co-regulatory molecule modulating inflammatory responses [33]. In chronic gastritis, particularly *H. pylori*-associated gastritis, sustained immune activation may drive CD276 expression as part of regulatory mechanisms attempting to limit inflammatory tissue damage. ROC analysis stratified by inflammatory status demonstrated that CD276 maintained good diagnostic performance even when comparing cancer to gastritis controls (AUC 0.765), though somewhat reduced compared to cancer versus normal controls (AUC 0.879). This indicates that while inflammatory confounding is present, CD276 retains capacity to distinguish malignancy from benign inflammation in routine clinical scenarios.

The correlation analysis provides crucial insight into the clinical relevance of CD276 and DKK3, extending their role beyond simple diagnosis to indicators of tumor aggression. The most consistent and biologically intuitive finding involves the inverse correlation of DKK3 with preoperative weight loss (rho = −0.544, *p* < 0.001). DKK3 is a protein implicated in tumor suppression and cellular homeostasis. Its significant decrease is a hallmark of advanced GC, and this strong negative correlation with patient catabolism (weight loss) suggests that the loss of DKK3 may be tightly linked to the systemic, cachexia-inducing effects of advanced gastric cancer. This makes DKK3 a promising serological marker for monitoring the overall metabolic burden and systemic progression of the disease.

Another very important aspect we would like to mention is that, in addition to the diagnostic and prognostic role these markers can play in malignant diseases, they can also be a therapeutic target [39,40].

Otherwise, CD276 may be an important target for monoclonal antibody or genetic therapy, as it is expressed in large quantities in tumor tissue compared to normal tissues [41].

To date, immunotherapy against CD276 includes the use of monoclonal antibodies, B7-H3-specific antibody-dependent cellular cytotoxicity (ADCC), B7-H3-specific antibody–drug conjugates (ADC), radionuclide radioimmunotherapy, genetically modified chimeric antigen receptor T cells (CAR-T), and bispecific antibodies directed against B7-H3 and CD3 [42].

On the other hand, increasing DKK levels through the use of demethylating agents, such as zebularine, or through the use of a CRISPR-based approach can reduce tumor cell proliferation and migration [43].

A study conducted on mice revealed that the intraperitoneal administration of an adenoviral vector carrying REIC/DKK-3 (Reduced Expression in Immortalized Cells/DKK3) induces a decrease in the dissemination and progression of gastric cancer cells in intraperitoneal nodules associated with gastric cancer [44]. An increase in the number of natural killer cells was also observed at this level of intraperitoneal tumors [45].

### Limitations

This study has several limitations that must be acknowledged. First, the primary analysis was conducted on a relatively small, two-center cohort of 40 gastric cancer patients and 40 healthy controls. This modest sample size limits the statistical power for subgroup analyses and may affect the generalizability of the findings to broader populations. Second, the case group was heavily skewed towards locally advanced disease (72.5% Stage III/IVA), which, while useful for correlative analysis, restricts our ability to determine the diagnostic efficacy of this biomarker panel for early-stage gastric cancer. The primary clinical value of serological biomarkers lies in early-stage detection, yet we had insufficient Stage I/II cases to perform robust stage-stratified analyses. Therefore, we cannot determine whether the CD276/DKK3 panel maintains diagnostic performance in early gastric cancer—a critical unresolved question requiring future studies in screening populations. Finally, the follow-up period for the patient cohort was insufficient, resulting in a very low number of events (deaths and recurrences). This rendered the prognostic survival analysis (both OS and DFS) statistically unstable and underpowered, making it impossible to confirm the independent prognostic value of CD276 or DKK3. Therefore, while the diagnostic potential of the panel is high, its prognostic utility remains undetermined, and a larger, multi-center validation study with extended follow-up is required.

## 5. Conclusions

In a cohort consisting predominantly of locally advanced gastric cancer, CD276 demonstrates excellent standalone diagnostic accuracy; however, the addition of DKK3 does not demonstrate a statistically significant improvement in the diagnostic accuracy. However, performance in early-stage gastric cancer remains undetermined due to the advanced-stage composition of our cohort. Validation studies in populations with higher prevalence of early-stage disease are essential to determine whether this panel can fulfill the primary unmet need: early detection when curative treatment is possible. These findings provide proof-of-concept and biological plausibility but do not yet constitute evidence for clinical diagnostic application.

## Figures and Tables

**Table 1:** Inclusion and exclusion criteria for Group 1 and Group 2.

	Inclusion Criteria	Exclusion Criteria
Group 1 (the group of patients with gastric cancer)	Patients who: have been diagnosed with anatomopathologically confirmed gastric cancer using the World Health Organization (WHO) classification system; underwent curative or palliative gastric resection in the aforementioned centers between 21 September 2023, and 28 February 2025 agreed to be included in the study and signed the consent form had complete clinical data Administration of neoadjuvant chemotherapy or tumor stage was not a criterion for exclusion from the study.	Patients who: did not agree to be included in the study are known to have immunosuppressive diseases such as HIV infection are known to have recent or chronic acute inflammatory diseases could not be contacted during the study period had a history of other malignant diseases or were diagnosed with another concomitant malignant disease
Group 2 (the control group)	People who: benefited from the exclusion of a malignant gastric pathology by performing an esophagogastroduodenoscopy (EGD) with gastric mucosa biopsy between 21 September 2023, and 28 February 2025 have no history of other malignant disease agreed to participate in the study and signed the consent form	People who: suffer from chronic diseases such as liver cirrhosis or heart failure are known to have immunosuppressive diseases such as HIV infection had incomplete clinical data

**Table 2:** Demographic and clinical characteristics of patients from Group 1.

Characteristics	Gastric Cancer (*n* = 40)
Age, years	
Mean ± SD	65.83 ± 8.47
Median (IQR)	66 (61.25–70.75)
Sex, *n* (%)	
Male	31 (77.5%)
Female	9 (22.5%)
Area of residence, *n* (%)	
Urban	22 (55%)
Rural	18 (45%)
Preoperative weight loss (kg)	
Mean ± SD	6.18 ± 3.91
Median (IQR)	5 (3–9.75)
Smoking status, yes, *n* (%)	26 (65%)
Alcohol consumption, yes, *n* (%)	20 (50%)
Tumor Location, *n* (%)	
Antral	27 (67.5%)
Cardia	4 (10%)
Gastric body	6 (15%)
Fornix	1 (2.5%)
Lesser curvature	1 (2.5%)
Pyloric	1 (2.5%)
Neoadjuvant treatment, yes, *n* (%)	16 (40%)
DKK3	
Mean ± SD	92.5 ± 27.4
Median (IQR)	86.46 (70.39–115.49)
CD276	
Mean ± SD	60.06 ± 39.53
Median (IQR)	59.77 (22.74–90.16)
Preoperative CEA Levels	
Mean ± SD	108.96 ± 333.25
Median (IQR)	7.3 (5.15–12.03)
Preoperative CA 19-9 Levels	
Mean ± SD	108.8 ± 204.09
Median (IQR)	66.5 (37.25–107.5)
Extent of Lymphadenectomy	
D1	-
D1+	12 (30%)
D2	28 (70%)
Histopathological subtypes, *n* (%)	
Intestinal-type adenocarcinoma	34 (85%)
Signet ring cell carcinoma	6 (15%)
Tumor differentiation grade, *n* (%)	
G1	3 (7.5%)
G2	27 (67.5%)
G3	10 (25%)
Resection margins, *n* (%)	
Positive	2 (5%)
Negative	38 (95%)
Perivascular Invasion, yes, *n* (%)	27 (67.5%)
Perineural invasion, yes, *n* (%)	23 (57.5%)
Tumor Size	
Mean ± SD	4.26 ± 1.58
Median (IQR)	4.4 (3–5.43)
Ratio of Metastatic Lymph Nodes to Total Lymph Nodes Harvested	
Mean ± SD	0.34 ± 0.26
Median (IQR)	0.35 (0.7–0.59)
Presence of Lymphatic Invasion (N Staging)	
N0	6 (15%)
N1	9 (22.5%)
N2	5 (12.5%)
N3a	13 (32.5%)
N3b	7 (17.5%)
Peritoneal Fluid Cytology, yes, *n* (%)	17 (42.5%)
Tumor Depth (T stage), *n* (%)	
T1	1 (2.5%)
T2	12 (30%)
T3	13 (32.5%)
T4a	13 (32.5%)
T4b	1 (2.5%)
M Stage, *n* (%)	
M0	36 (90%)
M1	4 (10%)
Death, yes, *n* (%)	6 (15%)
Tumor stage, *n* (%)	
IA	1 (2.5%)
IB	3 (7.5%)
IIA	5 (12.5%)
IIB	6 (15%)
IIIA	6 (15%)
IIIB	12 (40%)
IIIC	3 (7.5)
IVA	4 (10%)
*Helicobacter pylori* status, yes, *n* (%)	25 (62.5%)

SD, standard deviation; IQR, interquartile range; CEA, Carcinoembryonic Antigen; CA 19-9, Carbohydrate Antigen.

**Table 3:** Baseline characteristics of individuals in Group 2.

Characteristics	No Gastric Cancer (*N* = 40)	With Gastritis (*N* = 15)	Without Gastritis (*N* = 25)	*p*-Value
Age, years				0.031 *
Mean ± SD	62 ± 12.03	62.3 ± 9.32	53.8 ± 12.5	
Median (IQR)	62 (29–74)	63 (56.5–69)	59 (43–62)	
Sex, *n* (%)				0.870 **
Male	22 (55%)	8 (53%)	14 (56%)	
Female	18 (45%)	7 (47%)	11 (44%)	
Area of residence, *n* (%)				0.864 **
Urban	26 (65%)	5 (33%)	9 (36%)	
Rural	14 (35%)	10 (67%)	16 (64%)	
*H. pylori* status, *n* (%)				0.641 **
Yes	8 (20%)	4 (27%)	4 (16%)	
No	30 (75%)	10 (67%)	20 (80%)	
unspecified	2 (5%)	1 (7%)	1 (4%)	
DKK3				0.804 *
Mean ± SD	92.5 ± 27.4	123 ± 23.9	120 ± 19.5	
Median (IQR)	86.46 (70.39–115.49)	116 (107–133)	112 (108–132)	
CD276				0.003 *
Mean ± SD	60.06 ± 39.53	24.3 ± 15.6	15.3 ± 18.4	
Median (IQR)	59.77 (22.74–90.16)	20.6 (18.3–24.3)	8.16 (6.08–13.0)	

* Mann–Whitney U test, ** χ^2^ test.

**Table 4:** Diagnostic performance of CD276 stratified by control inflammatory status.

Comparison	AUC (95%CI)	N (Cases/Controls)	Sensitivity	Specificity
Cancer vs. Normal controls	0.879 (0.8–0.96)	40/25	84.0%	80.0%
Cancer vs. Gastritis controls	0.765 (0.64–0.89)	40/15	67.5%	86.7%
Cancers vs. All controls	0.837 (0.744–0.913)	40/40	77.4%	72.1%

**Table 5:** Univariable and multivariable Cox regression analysis of disease-free survival in gastric cancer patients *.

	Univariable Analysis		Multivariable Analysis	
	HR (95% CI)	*p*-Value	HR (95% CI)	*p*-Value
CD276 (ref. low risk)	364 × 10^6^ (0-Inf)	0.99	7 × 10^6^ (0-Inf)	0.995
DKK3 (ref. low risk)	3.53 (0.41–30.35)	0.25	0.61 (0.06–5.96)	0.67
T stage (ref. T1)				
T2	1 (0-Inf)	1.0	0 (0-Inf)	0.996
T3	41 × 10^6^ (7 × 10^6^–230 × 10^6^)	<0.001	7.01 (1.01–48.8)	0.04
T4a	72 × 10^6^ (14 × 10^6^–367 × 10^6^)	<0.001	4.36 (0.85–22.5)	0.078
T4b	764 × 10^6^ (76 × 10^6^–766 × 10^7^)	<0.001	8.01 (0.81–78.7)	0.074
Ratio nodes	40.12 (0.73–2208.41)	0	0.33 (0.01–12.5)	0.547
M stage (ref. M0)	81.53 (1.1–6061.86)	0	0.54	0.77

* This analysis is severely underpowered (*n* = 6 events); results are exploratory and hypothesis-generating only.

## Data Availability

The data presented in this study are available on request from the corresponding author due to data protection.
